# Silencing of *ROT2*, the Encoding Gene of the Endoplasmic Reticulum Glucosidase II, Affects the Cell Wall and the *Sporothrix schenckii*–Host Interaction

**DOI:** 10.3390/jof8111220

**Published:** 2022-11-18

**Authors:** Luz A. López-Ramírez, Iván Martínez-Duncker, Anayeli Márquez-Márquez, Ana P. Vargas-Macías, Héctor M. Mora-Montes

**Affiliations:** 1 Departamento de Biología, División de Ciencias Naturales y Exactas, Campus Guanajuato, Universidad de Guanajuato, Noria Alta s/n, col. Noria Alta, C.P., Guanajuato 36050, Mexico; 2 Laboratorio de Glicobiología Humana y Diagnóstico Molecular, Centro de Investigación en Dinámica Celular, Instituto de Investigación en Ciencias Básicas y Aplicadas, Universidad Autónoma del Estado de Morelos, Cuernavaca 62209, Mexico

**Keywords:** fungal cell wall, protein glycosylation, *N*-linked glycans, *O*-linked glycans, virulence, phagocytosis, innate immune sensing

## Abstract

*Sporothrix schenckii* is a member of the *Sporothrix* pathogenic clade and one of the most common etiological agents of sporotrichosis, a subcutaneous fungal infection that affects both animal and human beings. Like other fungal pathogens, the *Sporothrix* cell wall is composed of structural polysaccharides and glycoproteins that are covalently modified with both *N*-linked and *O*-linked glycans. Thus far, little is known about the *N*-linked glycosylation pathway in this organism or its contribution to cell wall composition and interaction with the host. Here, we silenced *ROT2*, which encodes the catalytic subunit of the endoplasmic reticulum α-glucosidase II, a processing enzyme key for the *N*-linked glycan core processing. Silencing of *ROT2* led to the accumulation of the Glc_2_Man_9_GlcNAC_2_ glycan core at the cell wall and a reduction in the total content of *N*-linked glycans found in the wall. However, the highly silenced mutants showed a compensatory mechanism with increased content of cell wall *O*-linked glycans. The phenotype of mutants with intermediate levels of *ROT2* silencing was more informative, as they showed changes in the cell wall composition and exposure of β-1.3-glucans and chitin at the cell surface. Furthermore, the ability to stimulate cytokine production by human mononuclear cells was affected, along with the phagocytosis by human monocyte-derived macrophages, in a mannose receptor-, complement receptor 3-, and TLR4-dependent stimulation. In an insect model of experimental sporotrichosis, these mutant cells showed virulence attenuation. In conclusion, *S. schenckii ROT2* is required for proper *N*-linked glycosylation, cell wall organization and composition, and interaction with the host.

## 1. Introduction

*Sporothrix schenckii*, *Sporothrix brasiliensis*, *Sporothrix globosa*, and *Sporothrix luriei* belong to the pathogenic clade of the *Sporothrix* genus and are the main causative agents of both animal and human sporotrichosis [1,2,3]. This infectious disease occurs as a benign acute or chronic granulomatous subcutaneous mycosis that rarely becomes a disseminated infection and usually requires immunosuppression to spread and compromise internal organs [4,5]. Thus far, *S. schenckii* is the species most thoroughly studied in both the basic and clinical aspects [6] and is distributed worldwide [7], while *S. brasiliensis* has recently attracted attention because of the caused zoonotic outbreak in Brazil that is changing the current paradigm of the infectious agent transmission [8,9]. Like other fungal pathogens, these species show thermodimorphism, growing as conidium-producer mycelia in the environment and switching to yeast-like cells when affecting the host tissues; thus, the latter has been traditionally considered the infective form [3,10,11].

The study of the fungal cell wall is an attractive and active research area because it provides basic information about the dynamic metabolism that supports the constant modification and reorganization of this plastic component of the fungal cell and also because it gathers valuable information about virulence factors and other fungal traits that help the pathogen to interact with the host cell and tissues [12,13,14]. Moreover, it is a source of molecules that can be used as molecular targets for the development of new antifungal drugs [15,16] and contains molecules that induce and modulate host immunity [3,12,13,17,18]. In *S. schenckii*, the cell wall is composed of two layers: the inner layer contains chitin and β-1,6-, β-1,4-, and β-1,3-glucans [3,19,20], and the outer layer is composed of glycoproteins modified with *N*-linked and *O*-linked glycans and peptidorhamnomannan, which is a complex of molecules with a wide molecular weight range, containing 57% of mannose, 33.5% of rhamnose [19,21,22], and over 300 different polypeptide chains [23].

Protein glycosylation is a ubiquitous posttranslational modification found in both eukaryotic and prokaryotic cells and involves the enzyme-driven covalent addition of monosaccharides or oligosaccharides to proteins [24]. In eukaryotic cells, including *S. schenckii,* the most common glycosylation processes are *N*-linked glycosylation, *O*-linked glycosylation, and the addition of glycosylphosphatidylinositol anchors [3]. The *N*-linked glycosylation pathway is divided into two stages, one performed in the endoplasmic reticulum (ER) and the second one performed in the Golgi complex, the former being highly conserved among eukaryotes, with diverse steps carried out in the Golgi compartment, and responsible for the species-specific *N*-linked glycans [25,26,27]. Thus far, most of the components of this metabolic pathway have been already predicted by bioinformatics means within the *S. schenckii* genome, but only a handful of them have been experimentally confirmed [3,11,28,29,30,31,32,33,34]. Similar to other fungal species, it is thought that the *N*-linked glycan core Glc_3_Man_9_GlcNAc_2_ attached to dolichol-phosphate is synthesized in the ER, then transferred to the canonical sequon Asp-X-Ser/Thr (X = any amino acid except Pro) of nascent proteins, and finally processed by α-glycosidases before transportation to the Golgi complex [26,27]. The α-glucosidase II belongs to this processing step and acts after α-glucosidase I has trimmed the outermost α-1,2-glucose from the *N*-linked core, removing the two remaining glucose units [35]. This hydrolytic remotion of glucose is not only essential for the further processing of the *N*-linked glycan core by the ER α-1,2-mannosidase but also relevant to generate the proper substrate for full elongation of the *N*-linked glycan outer chain by Golgi-resident mannosyltransferases [26,36,37]. Moreover, the α-glucosidase II also participates in the ER quality control system for misfolded proteins, where, in collaboration with chaperones and the UDP-glucose glycoprotein glucosyltransferase, it helps in labeling misfolded glycoproteins for proteasomal degradation [38]. In most eukaryotic systems, the α-glucosidase II enzyme is a heterodimer of the alpha and beta subunits, which have the catalytic site and the retention signals for ER retention, respectively [35].

In *S. schenckii*, it was previously demonstrated that the cells have this enzyme activity and the encoding gene of the catalytic subunit is *ROT2*. However, we currently do not have reports assessing the contribution of this gene to fungal fitness. Therefore, here, to get insight into the contribution of α-glucosidase II to cell wall composition and the *Sporothrix*–host interaction, we generated *ROT2*-silenced strains and analyzed the interaction of these cells with human primary peripheral blood mononuclear cells (PBMCs), monocyte-derived macrophages, and the virulence in an insect model of disseminated sporotrichosis.

## 2. Materials and Methods

### 2.1. Strains and Culturing Conditions

*S. schenckii* ATCC MYA-4821 [39] was used to generate the silenced strains analyzed in this work and is referred to as the wild-type (WT) strain. The YPD medium, pH 4.5 (1% (*w*/*v*) yeast extract, 2% (*w*/*v*) gelatin peptone, and 3% (*w*/*v*) dextrose), and incubation at 28 °C were used to maintain fungal cells, while *Agrobacterium tumefaciens* AGL-1 was grown at 28 °C in Luria–Bertani broth (0.5 (*w*/*v*) yeast extract, 1% (*w*/*v*) gelatin peptone, and 1% (*w*/*v*) NaCl). Bacteria harboring the binary vector were selected by growing in presence of 100 μg mL^−1^ ampicillin and 100 μg mL^−1^ kanamycin. Conidia production was carried out in YPD plates, pH 4.5, at 28 °C for 7 days, and were harvested by surface scratching, as described [30]. These cells were further incubated in YPD broth, pH 4.5, at 28 °C, and 120 rpm for 48 h, and mycelia were harvested by vacuum-assisted filtering, using a 5 μm pore size nylon membrane. Yeast-like cells were obtained growing conidia in YPD broth, pH 7.8 for 4 days at 37 °C and 120 rpm, as described [20], and harvested by centrifuging at 5000× *g* and 4 °C for 5 min. In all cases, cells were washed three times with deionized water and kept at −20 °C until used. In some experiments, cells were heat-killed by incubating at 60 °C for 2 h, and loss of cell viability was confirmed by incubating on YPD plates, pH 4.5, at 28 °C for 5 days [20]. Fungal cells containing the binary vector were selected on YPD plates, pH 4.5, added with 400 mg mL^−1^ hygromycin B, as reported [11,40,41].

### 2.2. Sporothrix schenckii ROT2 Silencing

A 221-bp fragment of the *ROT2* open reading frame (GenBank accession code JQ390409) was amplified by PCR using genomic DNA and the primer pair 5′-CTCGAGCTACAACATCCTGCCCGAGT and 5′-AAGCTTCCTCGTTGTAGCGTTCTTTCC (underlined sequences are added recognition sites for XhoI and HindIII, respectively), and this amplicon was cloned into the pSilent-1 XhoI and HindIII sites [42], generating pSilent-1-ROT2-sense. A second primer pair, with the same sequence but with adaptors for StuI and BglII, was used to amplify by PCR the amplicon that was cloned into the pSilent-1-ROT2-sense StuI and BglII sites, generating pSilent-1-ROT2-sense-antisense. Then, using this vector as a template and the primer pair 5′-CTGCAGATGCCAGTTGTTCCCAGTGATC and 5′-GAGCTCCCTCTAAACAAGTGTACCTGTGCATT (underlined sequences are added recognition sites for PstI and SacI, respectively), a 2626-bp amplicon was amplified by PCR, corresponding to the *Aspergillus nidulans trpC* promoter, the multicloning site, and the *A. nidulans trpC* terminator [11,40]. This PCR fragment was cloned into the pBGgHg PstI and SacI sites [43], generating the binary plasmid pBGgHg-ROT2. The *A. tumefaciens* AGL-1 was transformed with this construction, and then cells were prepared for fungal transformation by growing bacteria in a minimal medium (0.34 M K_2_HPO_4_, 0.16 M NaH_2_PO_4_, 0.37 M NH_4_Cl, 0.24 MgSO_4_, 0.04 M KCl, 1.8 mM CaCl_2_, 0.18 mM FeSO_4_, pH 7.0 adjusted with 1 N HCl) added with 200 μM acetosyringone (Sigma-Aldrich, San Luis, MO, USA) for 4.5 h at 28 °C and orbital shaking at 250 rpm [41]. Bacteria and conidia interactions were performed as previously reported [41], bacteria were killed by incubating the cell interactions on YPD, pH 4.5, added with 400 mg mL^−1^ hygromycin B and 200 μM cefotaxime, and incubated for 72 h at 28 °C. The transformant cells were subjected to five monoconidial passages in YPD, pH 4.5, added with 400 mg mL^−1^ hygromycin B, and three rounds of dimorphism induction in YPD, pH 7.8, added with 400 mg mL^−1^ hygromycin B [41]. The binary plasmid insertion within the *S. schenckii* genome was confirmed by PCR, using the primer pair 5′-GGCGACCTCGTATTGGGAATC and 5′-CTATTCCTTTGCCCTCGGACGAG-3′, as reported [11,40].

### 2.3. Quantification of Gene Expression and the Binary Plasmid Insertional Events

Total RNA was extracted as described elsewhere [44], and cDNA was synthesized using oligo(dT)20 primer [45], purified by chromatography [46], quantified in a NanoDrop 2000 (Thermo Fisher Scientific, Waltham, MA, USA), and used in qPCR reactions containing SYBR Green PCR Master Mix and performed in a thermocycler StepOne Plus (Life Technologies, Carlsbad, CA, USA) [34]. The primer pair used in the reactions was 5′-CTACAACATCCTGCCCGAGT and 5′-CCTCGTTGTAGCGTTCTTTCC, which amplified the *ROT2* region cloned into pBGgHg-ROT2. The same strategy was used to assess the number of the binary plasmid insertional events but using genomic DNA instead. Both methodologies were analyzed in the StepOne software V 2.2 (Life Technologies), using the 2^−ΔΔCt^ method [47], the encoding gene for the ribosomal protein L6 for data normalization, and the WT strain as the reference condition. The primer pair 5′-ATTGCGACATCAGAGAAGG and 5′-TCGACCTTCTTGATGTTGG was used for amplification of the encoding gene for the ribosomal protein L6 [45].

### 2.4. Measurement of α-Glucosidase Activity

The α-glucosidase activity was measured as previously described [44,48], using 4-methylumbelliferyl-α-D-glucopyranoside (Sigma-Aldrich) as substrate. Yeast-like cells were grown for 4 days at 37 °C in YPD broth, pH 7.4, pelleted by centrifuging, and disrupted with glass beads in an MSK cell homogenizer (Braun, Melsungen, Germany). Cell walls and debris were pelleted by centrifuging, and the supernatant was saved and kept at −20 °C until used. The enzyme reactions were prepared in a volume of 200 μL containing 200 μg protein, 40 μM 4-methylumbelliferyl-α-D-glucopyranoside, and 50 mM sodium phosphate buffer, pH 7.0, and were incubated at 37 °C for 60 min. Reactions were terminated by adding 3.3 mL of 50 mM glycine-NaOH buffer, pH 11.0, and the free 4-methylumbelliferone was measured in an LS-5B spectrofluorometer (Perkin-Elmer, Waltham, MA, USA), with excitation and emission wavelength set at 350 and 440 nm, respectively. Total activity was expressed as nanomoles of 4-methylumbelliferone released per min per total protein. To differentiate between α-glucosidase I and α-glucosidase II activities, the enzyme reactions were performed in presence of 10 μM castanospermine (Sigma-Aldrich) [32,45].

### 2.5. Analysis of Cell Wall N-Linked and O-Linked Glycans

Yeast-like cells grown as indicated were quantified and adjusted to 1 × 10^9^ yeast-like cells mL^−1^. For *N*-linked glycans trimming, 1 mL aliquots were pelleted by centrifuging, cells were resuspended in 3 mM NaOAc, 25 U endoglycosidase H (New England Biolabs, Ipswich, MA, USA) was added, and the preparations were incubated at 37 °C for 24 h [49,50]. For the case of *O*-linked glycans, 1 × 10^9^ yeast-like cells were suspended in 0.1 N NaOH and incubated overnight at room temperature with gentle orbital shaking [49,51]. In both cases, cell preparations were neutralized with either 1 N HCl or 1 N NaOH, centrifuged to pellet cells, and supernatants were saved, lyophilized, and stored at −20 °C until used. The phenol-sulfuric-acid method was used to quantify the total sugar content [52] for analysis of the *N*-linked glycan core by high-performance anion-exchange chromatography with pulsed amperometric detection (HPAEC-PAD). Samples were filtrated in an Amicon Ultra-0.5 Centrifugal Filter Unit, and the eluted material was separated in a Dionex system (Thermo Fisher Scientific) equipped with a CarboPac PA-100 column (4.6 × 250 mm and a guard column. Separation conditions were a linear gradient of 10–100 mM sodium acetate in 100 mM NaOH at a flow rate of 0.8 mL min^−1^. The pure Glc_2_Man_9_GlcNAc_2_ (Sigma-Aldrich) was used as standard. Alternatively, the *N*-linked glycan core was digested with α-glucosidase from *Bacillus stearothermophilus* (Megazyme, Wicklow, Ireland) [53], and free glucose was quantified by HPAEC-PAD, as elsewhere described [49].

### 2.6. Cell Wall Analysis

Cell homogenates were prepared as described in Section 2.4, using yeast-like cells and an MSK cell homogenizer. Cell walls were pelleted and subject to a cleansing protocol that included six washes with deionized water, incubation with hot SDS, β-mercaptoethanol, and NaCl, as described [36]. Then, samples were acid hydrolyzed with 2 M trifluoroacetic acid and analyzed by HPAEC-PAD as described [54]. Cleansed cell walls were alkali-hydrolyzed before protein quantification with the Pierce BCA Protein Assay (Thermo Fisher Scientific) [36]. For cell wall porosity analysis, the yeast-like cells were adjusted to a concentration of 1 × 10^8^ cell mL^−1^, aliquots containing 1 mL were centrifuged, and cells were suspended in 10 mM Tris-HCl, pH 7.4 (buffer A), buffer A plus 30 μg mL^−1^ poly-L-lysine (MW 30–70 kDa, Sigma-Aldrich) or buffer A plus 30 μg mL^−1^ diethylaminoethyl-dextran (MW 500 kDa, Sigma-Aldrich) [55]. After incubating the cell preparations for 30 min at 28 °C and gentle shaking, these were centrifuged, and the supernatants were saved and used to measure the absorbance at 260 nm. The 100% porosity was normalized to the readings obtained with cells incubated with poly-L-lysine, and from this, the relative cell wall porosity to diethylaminoethyl-dextran was calculated [55]. To assess the cells’ ability to bind Alcian blue, yeast-like cells were adjusted at an O.D._600nm_ of 0.2 in deionized water. One-microliter aliquots were pelleted, and cells were suspended in 1 mL of Alcian blue (Sigma-Aldrich; 30 μg mL^−1^, in 0.02 M HCl, pH 3.0) and incubated for 10 min; cells were pelleted by centrifuging, and the supernatant was used to measure the absorbance at 620 nm. The quantification of dye bound to cells was performed as previously reported [56].

To assess β-1,3-glucan and chitin localization within the cell wall, these polysaccharides were labeled as previously described [57,58]. Yeast-like cells were incubated with 5 μg mL^−1^ IgG Fc-Dectin-1 chimera for 40 min at room temperature [59] and then incubated with 1 μg mL^−1^ donkey anti-Fc IgG-FITC (Sigma-Aldrich) for 40 min at room temperature for β-1,3-glucan labeling [57]. Alternatively, cells were incubated with 0.5 mg mL^−1^ fluorescein isothiocyanate conjugated wheat germ agglutinin (WGA-FITC; Sigma-Aldrich) for 60 min at room temperature for chitin labeling [58]. In both cases, cells were examined under fluorescence microscopy with a Zeiss Axioscope-40 microscope (Carl Zeiss AG, Jena, Germany) and an Axiocam MRc camera. The fluorescence of 300 cells was collected using the software Adobe Photoshop™ CS6 and the formula: [(total of green pixels − background green pixels) × 100] total pixels^−1^ [60]. In both cases, the 100% polysaccharide exposure at the cell wall surface was the fluorescence associated with heat-killed cells.

### 2.7. Ethics Statement

The isolation and experimentation with primary human cells were approved by the Ethics Committee of Universidad de Guanajuato (reference CIBIUG-P12-2018). Only healthy adult volunteers participated in the study, detailed information about the study was provided, and written informed consent was signed before withdrawing venous blood samples. The procedures were conducted following the Declaration of Helsinki. Under the same reference code, the Ethics committee also approved the experimentation with animals included in this study.

### 2.8. Cytokine Stimulation Using Human Peripheral Blood Mononuclear Cells

Upon withdrawal of venous samples, PBMCs were isolated from EDTA-treated samples by density centrifuging in Histopaque-1077 (Sigma-Aldrich), as described [61]. Human cells were suspended in RPMI 1640 Dutch modification (Sigma-Aldrich) and seeded in 96-well microplates, at a cell concentration of 5 × 10^5^ human cells in 200 μL. To block specific immune receptors, human cells were pre-incubated for 60 min at 37 °C with 5% (*v*/*v*) CO_2_ with one of the following reagents: 200 μg mL^−1^ laminarin (Sigma-Aldrich) [62], 10 μg mL^−1^ anti-mannose receptor (MR) (Thermo-Fisher Scientific, MA5-44033), 10 μg mL^−1^ anti-TLR4 (Santa Cruz Biotechnology, Dallas, TX, sc-293072), 10 μg mL^−1^ anti-TLR2 (Thermo-Fisher Scientific, 16-9922-82), or 10 μg mL^−1^ anti-CD11b (Thermo Fisher, MA5-16528) [20,54,63]. Isotype-matched, irrelevant IgG1 antibodies (10 μg mL^−1^, Santa Cruz Biotechnology, Cat. No. sc-52003) were used as a control in experiments where MR and TLR4 were blocked; 10 μg mL^−1^ IgG2aκ (Thermo-Fisher Scientific, 14-4724-85) was used to control TLR2 blocking experiments, and 10 μg mL^−1^ IgG2 (R&D, Minneapolis, MN, USA, Cat. No. MAB9794) was used to control CD11b blocking assays. For fresh or preincubated PBMCs, 1 × 10^5^ yeast-like cells were included in each well, and plates were incubated for 24 h at 37 °C with 5% (*v*/*v*) CO_2_. The *Limulus* amebocyte lysate (Sigma-Aldrich) assays demonstrated that all reagents were LPS-free. Nonetheless, 5 μg mL^−1^ polymyxin B (Sigma-Aldrich) was included in all PBMC–fungus interactions [50,64]. In all cases, at the end of the incubation time, plates were centrifuged for 10 min at 1800× *g* at 4 °C, and supernatants were saved and kept at −20 °C until cytokine quantification. Tumor necrosis factor-alpha (TNFα), interleukin 6 (IL-6), and interleukin 10 (IL-10) were quantified by ELISA with Standard ABTS ELISA Development kits (Peprotech, Cranbury, NJ, USA), whereas interleukin 1β (IL-1β) was quantified with a DuoSet ELISA (R&D) (Minneapolis, MN, USA.) Mock interactions where only PBMCs were seeded in RPMI 1640 Dutch modification (Sigma-Aldrich) were included as a control in each plate.

### 2.9. Phagocytosis Assays

The human peripheral blood mononuclear cells were differentiated into macrophages by incubating with recombinant human granulocyte-macrophage colony-stimulating factor (Sigma-Aldrich), as described [60]. Yeast-like cells were incubated with 1 mg mL^−1^ Acridine Orange (Sigma-Aldrich) and washed with PBS, and cell concentration was adjusted at 3 × 10^7^ yeast-like cells mL^−1^ [65]. Cells were seeded in 6-well plates, at a macrophage: fungus ratio of 1:6, in a final volume of 800 μL DMEM medium (Sigma-Aldrich) and incubated for 2 h at 37 °C and 5% (*v*/*v*) CO_2_ [40,41]. Then, macrophages were washed twice with chilled PBS and stained with 1.25 mg mL^−1^ Trypan Blue, as an external fluorescence quencher [66]. Phagocytosis was analyzed by flow cytometry in a FACSCanto II equipped with a FACSDiva acquisition system (Becton Dickinson, Franklin Lakes, NJ, USA). Immune cells were gated, and 50,000 events were collected per sample, using the FL1 (green) and FL2 (red) channels for fluorescence signal acquisition, previously compensated with non-stained macrophages [11,40,65,66]. When required, macrophages were preincubated for 1 h at 37 °C and 5% (*v*/*v*) CO_2_ with 10 μg mL^−1^ of any of the following antibodies: anti-MR (Thermo-Fisher Scientific), anti-TLR4 (Santa Cruz Biotechnology), anti-TLR2 (Thermo-Fisher Scientific), anti-CD11b (Thermo Fisher), irrelevant IgG1 antibodies (Santa Cruz Biotechnology), or irrelevant IgG2aκ (Thermo-Fisher Scientific). Alternatively, cells were preincubated under the same conditions with 200 μg mL^−1^ laminarin (Sigma-Aldrich). All the interactions were performed in presence of polymyxin B 5 μg mL^−1^. Cells emitting fluorescence in the green channel were considered in the early stage of phagocytosis, cells emitting in both the green and red channels were considered at the intermediate stage of this immune event, and those emitting fluorescence only in the red channel were grouped in the late stage of the phagocytic process [11,40,65].

### 2.10. Virulence Assays

The experimental model of systemic sporotrichosis in *Galleria mellonella* larvae was used to assess fungal virulence. Larvae were from an in-house colony previously established [67] and were fed *ad libitum* on a corn bran and honey diet, as described [68]. Active larvae without body melanization and with a length of 1.2–1.5 cm were included in the assays. Upon sanitization with 70% (*v*/*v*) ethanol, inocula were injected in the last left pro-leg with a Hamilton syringe and a 26-gauge needle [67]. The fungal inoculum included 1 × 10^5^ yeast-like cells in 10 μL PBS. Larvae were kept in Petri dishes at 37 °C, and survival was monitored daily for 2 weeks. Chopped apple was included in the animal housing to keep hydration [68]. The absence of animal movement and extensive body melanization were taken as signs of insect death. Larvae were analyzed in groups that included 30 animals per experimental condition. As a control, one animal group was injected only with PBS. Colony-forming units were determined by decapitating both alive and dead animals; then, hemolymph was collected, serially diluted with sterile PBS, and incubated on YPD plates, pH 4.5, at 28 °C for 96 h. For the following parameters, groups of 10 animals were inoculated with the fungal strains as above described, incubated for 24 h at 37 °C, and decapitated to collect hemolymph. Phenoloxidase activity and cytotoxicity were assayed in cell-free hemolymph using 20 mM 3,4-dihydroxyDL-phenylalanine (Sigma-Aldrich) and the Pierce LDH Cytotoxicity Assay (Thermo Fisher Scientific), respectively [69,70]. Melanin production was quantified by measuring the absorbance at 405 nm of hemolymph, as elsewhere reported [71], while hemocyte levels were quantified in anticoagulated hemolymph [68].

### 2.11. Statistical Analysis

The GraphPad Prism 6 software was used for statistical analysis. The Mann–Whitney U test was used to analyze cytokine stimulation and phagocytosis by human cells. These experiments were carried out in duplicate with samples from eight healthy donors. Survival experiments were performed with a total of 30 larvae per group, and results were analyzed using the Log-rank test and were plotted in Kaplan–Meier survival curves. Other experiments were performed at least three times in duplicate, and the unpaired *t*-test was used to establish statistical significance. All data are represented as the mean and standard deviation. In all cases, the significance level was set at *p* < 0.05.

## 3. Results

### 3.1. Silencing of Sporothrix schenckii ROT2

To study the role of *ROT2* in the *S. schenckii* biology, in particular, during the host–fungus interplay, the WT strain ATCC MYA-4821 [39] was transformed with the binary vector pBGgHg-ROT2, using a previously standardized *A. tumefaciens* mediated-transformation methodology [11,40,41]. The backbone of this vector was pBGgHg [43], which confers resistance to hygromycin B and harbored a 221 bp fragment of the *ROT2* ORF (starts at the +276 position) cloned in sense and antisense orientations. Following the monoconidial passages described in the Materials and Methods to enrich transformed nuclei, the pBGgHg-ROT2 insertion within the *Sporothrix* genome was confirmed by PCR, amplifying the *Escherichia coli hph* gene found in pBGgHg-ROT2, and six colonies were selected, named HSS33-HSS38, for *ROT2* expression analysis. In parallel, the WT strain was *A. tumefaciens* mediated-transformed with the empty pBGgHg vector, and after transformed nuclei enrichment, two colonies, named HSS31 and HSS32, were selected and used as controls in this study.

**Figure 1:**
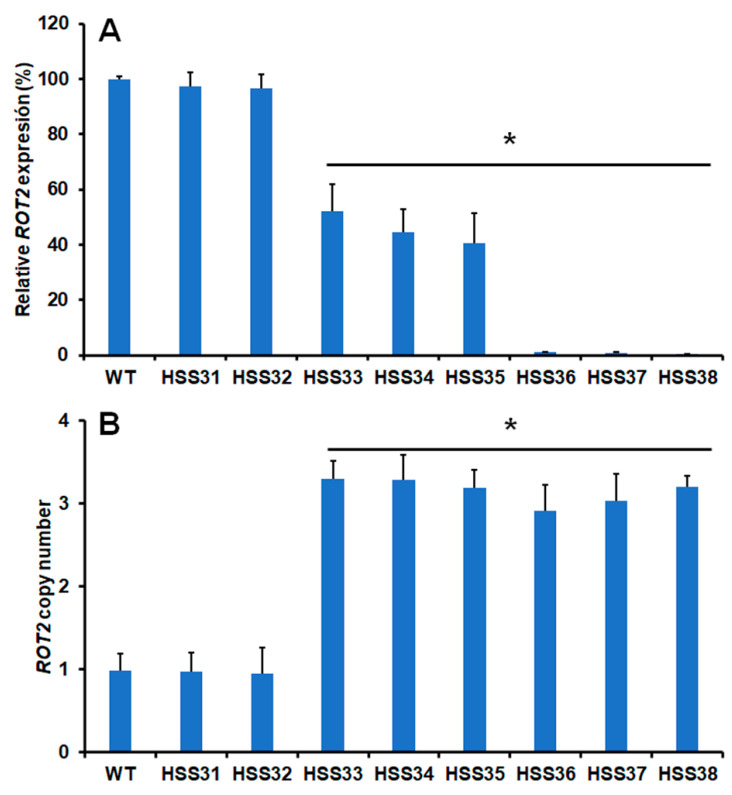
*ROT2* expression and analysis of binary vector insertional events in *Sporothrix schenckii* wild-type and mutant strains. In (**A**), total RNA was isolated from the different mutant strains and used in RT-qPCR reactions to amplify a specific part of the *ROT2* open reading frame. In (**B**), genomic DNA was isolated from the different strains and used in qPCR reactions that amplified the same DNA fragment used for gene expression analysis. In both cases, the amplification of the gene encoding the ribosomal protein L6 was used for data normalization. Data are means ± SD of three independent experiments performed in duplicates. * *p* < 0.05 when compared to the WT, HSS31, or HSS32 strains.

The *ROT2* expression was analyzed by RT-qPCR, and data were normalized using the *ROT2* gene expression of the WT strain and the expression of the encoding gene for the ribosomal protein L6, as reference strain and non-variable gene expression, respectively [45]. The *ROT2* expression in the WT and control strains HSS31 and HSS32 was similar, with no significant changes, minimizing the effect of the vector backbone on the *ROT2* expression (Figure 1A). For the case of mutants transformed with pBGgHg-ROT2, three strains showed an intermediate gene silencing (47.9 ± 9.9%, 55.4 ± 8.1%, and 59.3 ± 10.6% for HSS33, HSS34, and HSS35, respectively) and the remaining three were considered as mutants with a high degree of *ROT2* silencing (98.9 ± 0.1%, 99.2 ± 0.3%, and 99.6 ± 0.1% for HSS36, HSS37, and HSS38, respectively; Figure 1A). The binary vector randomly integrates within the fungal genome, and multiple insertional events have been previously reported when used to transform *S. schenckii* [41]. Thus, the number of the pBGgHg-ROT2 insertional events was analyzed by qPCR. We amplified the same *ROT2* 221 bp fragment cloned in the binary plasmid; therefore, in one ectopic insertional event, we should expect three copies of this fragment within the fungal genome, two provided by the binary vector and one amplified from the native locus. One *ROT2* copy was amplified from the WT and control strains, while three copies were detected in all the strains transformed with pBGgHg-ROT2, confirming that the silenced strains had only one copy of the binary vector integrated into the genome (Figure 1B). The six *ROT2*-silenced mutants and the two control strains showed no obvious defect in cell and colony morphologies and were capable to perform dimorphism, similar to the WT strain. The eight transformed and the WT strains showed similar doubling times for both mycelia and yeast-like cells, suggesting no growth defect in the control and silenced strains (mycelia 3.5 ± 0.4 h vs. 3.7 ± 0.3 h and yeast-like cells 8.1 ± 0.3 h vs. 7.9 ± 0.5 h for WT and average doubling times of the eight transformed strains, respectively). Therefore, *ROT2* silencing did not affect *S. schenckii* growth and morphology.

### 3.2. α-Glucosidase Activity and Protein Glycosylation Are Affected in the Sporothrix schenckii ROT2-Silenced Mutant Strains

To assess the impact of *ROT2* silencing in the α-glucosidase activity, and therefore in the *N*-linked protein glycosylation, we first measured the enzyme activity with the non-specific fluorogenic substrate 4-methylumbelliferyl-α-D-glucopyranoside, which at neutral pH is processed by either the endoplasmic reticulum α-glucosidase I or α-glucosidase II [32,45,48]. The enzyme activity was significantly reduced in the strains HSS33, HSS34, and HSS35, those with an intermediate *ROT2* silencing (Table 1), and enzyme activity was even more reduced in the strains with a higher *ROT2* silencing level, named HSS36, HSS37, and HSS38 (Table 1). No differences were observed among strains with similar silencing levels. The control strains HSS31 and HSS32 showed similar glucosidase levels to those observed in the WT strain (Table 1). To have an indirect measurement of α-glucosidase II activity, we performed the reactions in the presence of castanospermine, an ER α-glucosidase I inhibitor [32,37,45]. Following this strategy, glucosidase activity was reduced by about half in the protein preparations from the WT cells, when compared to the measurements without castanospermine, and a similar trend was observed for the samples from the control strains HSS31 and HSS32 (Table 1). The silenced strains with intermediate *ROT2* silencing, HSS33-HSS35, showed a significant reduction in the castanospermine insensitive glucosidase activity, and this was almost abolished in the strains with high levels of *ROT2* silencing (HSS36-HSS38; Table 1). Thus, these data suggested that α-glucosidase II activity was negatively affected by the *ROT2* silencing.

**Figure 2:**
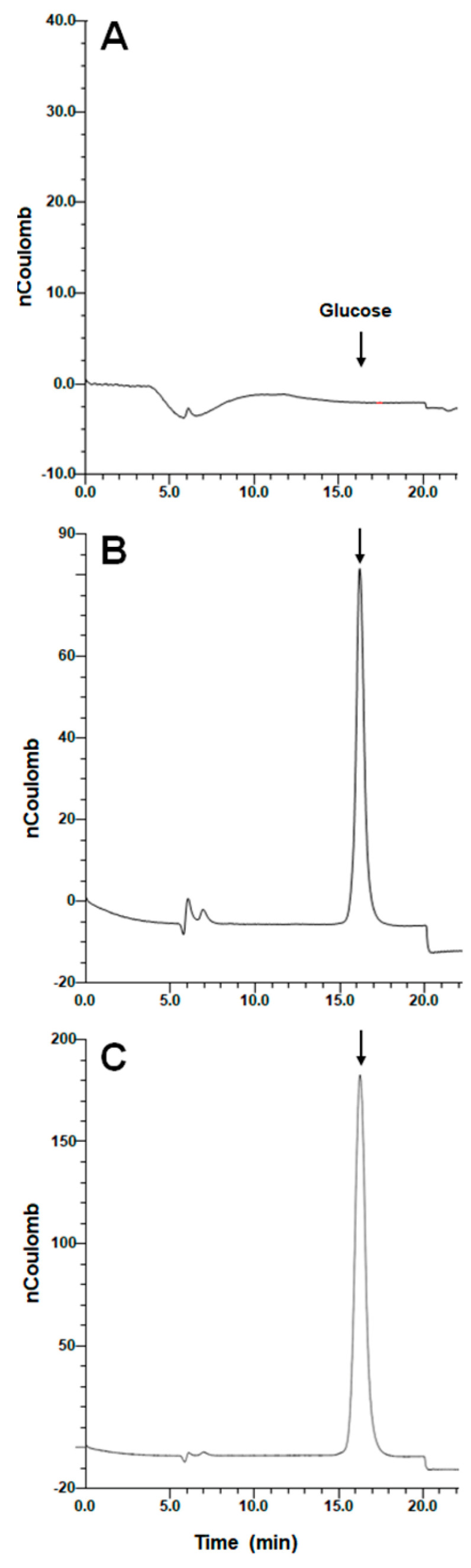
Detection of glucose in the *Sporothrix schenckii* cell wall *N*-linked glycan core. The *N*-linked glycans were trimmed from the yeast-like cell wall by incubating with 25 U of endoglycosidase H, the released *N*-linked glycans were filtrated in a 3 kDa-exclusion filtration unit, and the eluted material was hydrolyzed with recombinant α-glucosidase and analyzed by high-performance anion-exchange chromatography with pulsed amperometric detection. The arrow indicated the elution time of glucose. (**A**) A sample from the WT strain; (**B**) a sample from the *ROT2*-silenced mutant strain HSS34; (**C**) a sample from the *ROT2*-silenced mutant strain HSS38. Chromatograms are representative of experiments performed three times per duplicate.

It was previously demonstrated that following *Candida albicans* and *Candida tropicalis OCH1* disruption, a gene encoding for the first enzyme reaction in charge of the synthesis of the *N*-linked glycan outer chain, the *N*-linked glycan core was accumulated at the cell surface [72,73], and a similar observation was suggested for *C. albicans rot2*Δ null mutant cells [36]. Thus, we hypothesized that following *ROT2* silencing, the Glc_2_Man_9_GlcNAc_2_ oligosaccharide would be found at the *S. schenckii* cell surface. The detection and quantification of this *N*-linked glycan core were not possible in the samples from the WT strains or the control strains HSS31 and HSS32, but this oligosaccharide was found at similar levels in the *N*-linked glycans trimmed from the cell wall of mutants HSS33-HSS35 and was significantly higher in samples from the mutant strains HSS36-HSS38 (Table 1). A peak corresponding to GlcMan_9_GlcNAc_2_ oligosaccharide was also detected in sample preparations from mutants HSS34-HSS36 and represented the 24.2 ± 6.4% of the total oligosaccharide found in these samples. To confirm this was indeed the glucosylated *N*-linked glycan core, this was incubated with recombinant α-glucosidase with the ability to process α-1,3-glycosidic bonds [53], and released glucose was quantified by HPAEC-PAD. Again, the monosaccharide was not detected in samples from the WT or control strains, but this was found in the preparations from the silenced strains, with significantly higher levels in the mutant strains HSS36-HSS38 (Table 1). Representative chromatograms of samples from the WT, one mutant strain with intermediate *ROT2* silencing, and one with a high *ROT2* silencing degree are shown in Figure 2.

**Figure 3:**
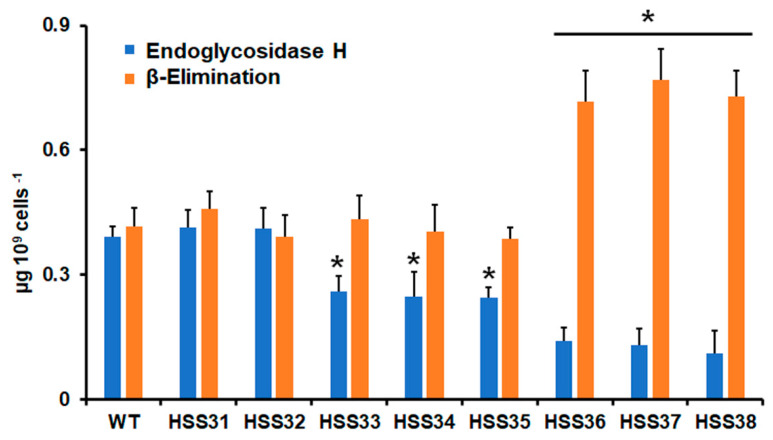
The *N*-linked and *O*-linked glycan content in the wild-type control and *ROT2*-silenced strains. The *N*-linked glycans were trimmed from yeast-like cell walls by incubating with endoglycosidase H, or *O*-linked glycans were removed by β-elimination. In both cases, the sugar content was quantified as described in Section 2.5. Data are represented as mean ± SD of three independent experiments performed in duplicates. * *p* < 0.05 when compared to WT, HSS31, or HSS32 cells.

When the total *N*-linked glycan content at the cell wall was quantified, no differences in the sugar content were found in the WT and the control strains HSS31 and HSSS32, but there was a significant difference in the *N*-linked glycan levels in the six *ROT2*-silenced strains, with the lowest *N*-linked glycan content observed in the mutant strains HSS36-HSS38 and intermediate levels in strains HSS33-HSS35 (Figure 3). When the *O*-linked glycans were removed by β-elimination [51] and the sugar content quantified, the WT, control, and mutant strains HSS33-HSS35 showed similar oligosaccharide content, and this was significantly increased in the silenced strains HSS36-HSS38 (Figure 3). Collectively, these data suggest that both *N*-linked and *O*-linked protein glycosylation was affected by *ROT2* silencing.

### 3.3. Silencing of ROT2 Affected the Sporothrix schenckii Cell Wall Composition

**Figure 4:**
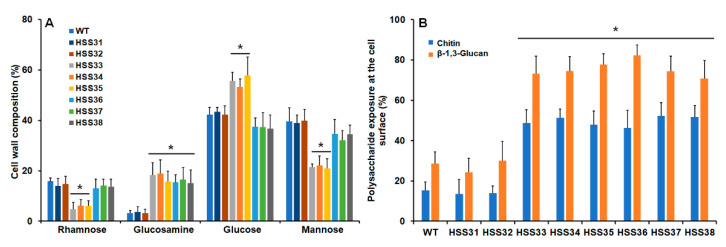
Cell wall analysis of *Sporothrix schenckii* wild-type, control, and *ROT2*-silenced mutant strains. In (**A**), the cell wall was isolated from yeast-like cells and acid hydrolyzed, and monosaccharides were identified and quantified by high-performance anion-exchange chromatography with pulsed amperometric detection. In (**B**), yeast-like cells were incubated with either fluorescein isothiocyanate (FITC) conjugated wheat germ agglutinin or IgG Fc-Dectin-1 chimera and anti-Fc IgG-FITC for chitin or β-1,3-glucan labeling, respectively, and the fluorescence associated to 300 cells was estimated. Data were normalized to the fluorescence observed in heat-killed cells, which corresponds to 100% in both cases. Data are represented as mean ± SD of three independent experiments performed in duplicates. * *p* < 0.05 when compared to WT, HSS31, or HSS32 cells.

Since the protein glycosylation was affected upon *ROT2* silencing, it was hypothesized that cell wall composition and organization were affected. The analysis of acid-hydrolyzed cell walls showed that walls isolated from the control strains HSS31 and HSS32 contained similar levels of rhamnose, mannose, glucosamine, and glucose to the WT strain (Figure 4A). Rhamnose and mannose are the building units found in *N*- and *O*-linked glycans, while glucosamine and glucose are the monomers of polysaccharides chitin and glucan, respectively [19,20,40,74]. The mutant strains with intermediate silencing, HSS33-HSS35, showed a significant reduction in the rhamnose and mannose and increased amounts of glucosamine and glucose (Figure 4A). However, the highly *ROT2*-silenced strains did not show a similar trend, observing only increased glucosamine levels (Figure 4A). The cell wall protein content was increased in all the silenced mutant strains (Table 2). Cell wall porosity and the ability to bind the cationic dye Alcian blue have been previously associated with protein glycosylation defects in *S. schenckii* [20,40,75], and the results reported here suggest the *ROT2* silencing led to defects in this posttranslational modification. The mutant strains with intermediate *ROT2* silencing levels showed increased wall porosity but lower ability to bind the dye, in line with the previous observations; however, the highly silenced strains (HSS36-HSS38) showed similar wall porosity and ability to bind Alcian blue to the WT strains (Table 2). We also analyzed the chitin and β-1,3-glucan organization within the cell wall by labeling the polysaccharides and observing the fluorescence intensity under fluorescent microscopy [57,58]. The inactivation of cells by heat artifactually exposed the inner wall components at the cell surface [20,58,76]; thus, the comparison of the fluorescence intensity of heat-killed with live cells allowed us to estimate how much of the polysaccharide is accessible on the cell surface [11,20,40,75,77]. Silencing of *ROT2* at intermediate or high levels positively affected the exposure of both chitin and β-1,3-glucan at the cell surface and to a similar extent (Figure 4B). The WT and control strains showed similar exposure levels of both polysaccharides (Figure 4B). Collectively, these data suggest that the *S. schenckii* cell wall suffered changes in the cell wall upon *ROT2* silencing.

### 3.4. Silencing of ROT2 Affected the Sporothrix schenckii–Human Innate Immune Cell Interactions

**Figure 5:**
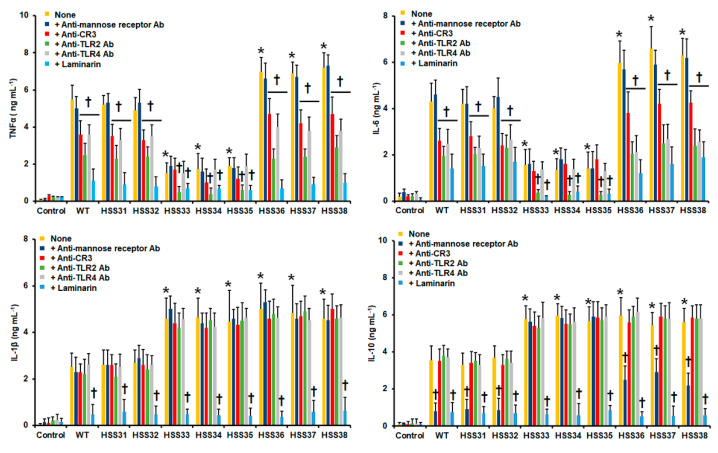
Cytokine stimulation by human peripheral blood mononuclear cells stimulated with *Sporothrix schenckii* wild-type, control, and *ROT2*-silenced strains. Human peripheral blood mononuclear cells were preincubated for 60 min with 200 μg mL^−1^ laminarin or 10 μg mL^−1^ of any of the following antibodies: anti-mannose receptor, anti-CR3 receptor, anti-TLR2, or anti-TLR4; then, they were coincubated with yeast-like cells for 24 h. In all cases, the interactions were centrifuged, supernatants collected, and cytokines quantified by ELISA. None refers to the system where human cells were preincubated with PBS and then challenged with the fungal cells. Ab, antibody. Control refers to wells where the human cells were preincubated only with PBS. Results are the media ± standard deviation from data generated with samples from eight donors, analyzed by duplicate. * *p* < 0.05, when compared with the cytokine level stimulated by WT strain. ^†^ *p* < 0.05, when compared with the same strain with no preincubation step.

Next, to assess the impact of the cell wall changes in the *ROT2*-silenced strains on the *S. schenckii* sensing by the host innate immunity, yeast-like cells were co-incubated with primary human PBMCs, and both pro-inflammatory and anti-inflammatory cytokines were measured as a readout of the interactions. The WT strain stimulated significant levels of TNFα, IL-1β, IL6, and IL-10 when compared to the control wells where human cells were preincubated with PBS, and the cytokine levels were similar when compared to the control strains HSS31 and HSS32 (Figure 5). For the case of these three strains, TNFα and IL-6 stimulation were dependent on the Complement Receptor 3 (CR3), TLR2, TLR4, and dectin-1, MR being dispensable for stimulation of these two cytokines (Figure 5). For the case of IL-1β, this was dectin-1 dependent, while IL-10 production depended on the stimulation of both TLR2 and dectin-1 (Figure 5). The *ROT2*-silenced strains with an intermediate *ROT2* silencing degree (HSS33-HSS35) stimulated lower TNFα and IL-6 levels than the control strain but higher IL-1β and IL-10 levels (Figure 5). Differently from the WT strain, TNFα and IL-6 stimulations were only TLR2 and dectin-1 dependent, and IL-1β and IL-10 production was solely dependent on dectin-1 (Figure 5). Contrary to the strains with middle *ROT2* silencing levels, the group of mutants with high levels of *ROT2* silencing stimulated higher TNFα and IL-6 levels, which were even higher than those observed with the WT strain, and cytokine stimulation showed the same receptor dependency as the WT strain (Figure 5). For the case of IL-1β production, the cytokine levels were similar to those observed with the strains HSS32-HSS35, with dectin-1-dependent stimulation, while for IL-10, similar levels were observed to those in the group of intermediate *ROT2* silencing, but in this case, the cytokine production was MR and dectin-1 dependent. As a control, the human cells were pre-incubated with irrelevant isotype-matched antibodies, and cells stimulated similar cytokine levels to PBMCs with no preincubating step (see Supplementary material, Figure S1).

**Figure 6:**
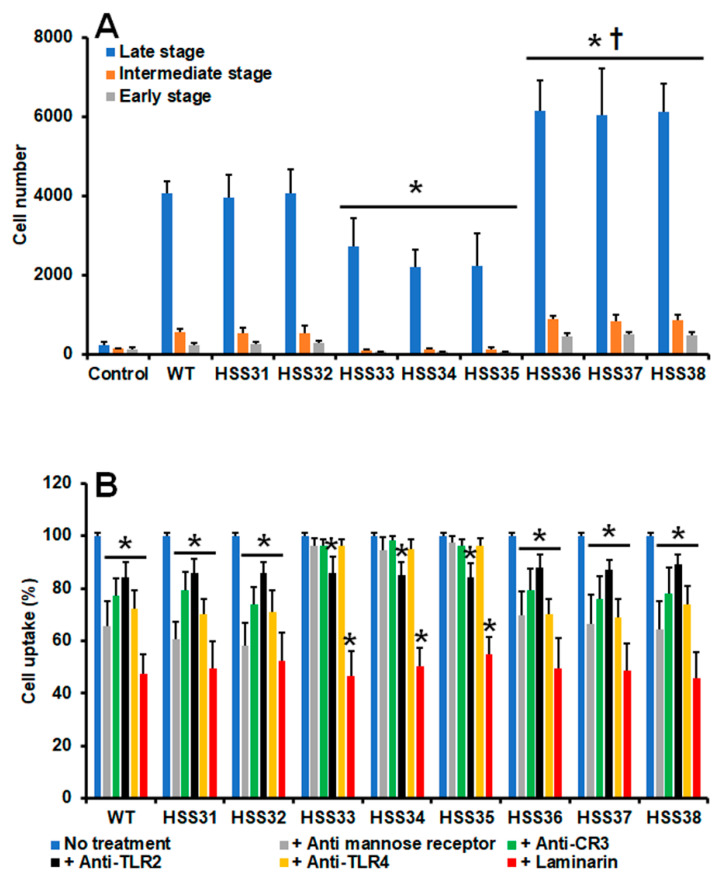
Phagocytosis of *Sporothrix schenckii* wild-type, control, and *ROT2*-silenced strains by human monocyte-derived macrophages. In (**A**), yeast-like cells were labeled with Acridine Orange, coincubated with human monocyte-derived macrophages at a macrophage–fungus ratio of 1:6, for 2 h at 37 °C and 5% (*v*/*v*) CO_2_. Human cells were gated by FACS, and 50,000 events, defined as a human cell interacting with at least one fluorescent fungal cell, were counted per sample. Control, macrophages interacting with no yeast-like cells. * *p* < 0.05 when compared to WT, HSS31, or HSS32 strains. ^†^ *p* < 0.05 when compared with HSS33, HSS34, or HSSS35 strains. In (**B**), experiments described in panel A were performed, but macrophages were previously preincubated with 10 μg mL^−1^ of any of the following antibodies: anti-mannose receptor, anti-CR3, anti-TLR2, or anti-TLR4. Alternatively, the human cells were preincubated with 200 μg mL^−1^ laminarin. In all cases, the interactions were performed in the presence of 5 μg mL^−1^ polymyxin B. No treatment refers to cells preincubated only with 5 μg mL^−1^ polymyxin B. Results correspond to cells in the late stage of phagocytosis. For all cases, 100% corresponds to the system with no treatment, and the absolute values were similar to those shown in panel A. CR3, complement receptor 3. * *p* < 0.05 when compared to the no treatment condition of the same strain. For both panels, data represent means ± SD from six donors assayed by duplicate.

The impact of *ROT2* silencing during the *S. schenckii*–macrophage interaction was also assessed, using human monocyte-derived macrophages and yeast-like cells. Fungal uptake by these immune cells was similar for WT and control strains in the early, intermediate, and late stages of the process (Figure 6A). For the case of the mutant strains with intermediate silencing levels (HSS33-HSS35), there was a significant reduction in fungal cells undergoing phagocytosis, but this trend was reverted in the highly silenced mutant strains, which were more readily phagocytosed than the WT, control, and intermediate-silenced mutant strains (Figure 6A). Control reactions where fungal cells were not included showed threshold fluorescent signals (Figure 6A). To explore the relevance of some of the pattern recognition receptors on the phagocytic process, blocking agents were used, similarly to the experiments performed with human PBMCs. The uptake of WT, control strains, and the highly silenced mutant strains was dependent on the engagement of MR, CR3, TLR2, TLR4, and dectin-1 with their corresponding ligands, the process being significantly dependent on dectin-1, and to a lesser extent of TLR2 (Figure 6B). However, for the case of strains HSS33-HSS35, the dependence of the phagocytic process on MR, CR3, and TL4 was lost, requiring only the engagement of dectin-1 and TLR2 with their corresponding ligands (Figure 6B). Control assays with irrelevant isotype-matching antibodies gave similar uptake levels to cells without the preincubation step. Collectively, these data indicate that *ROT2* silencing affected the interaction of *S. schenckii* with PBMCs and monocyte-derived macrophages.

### 3.5. Silencing of ROT2 Affected the Sporothrix schenckii Virulence

**Figure 7:**
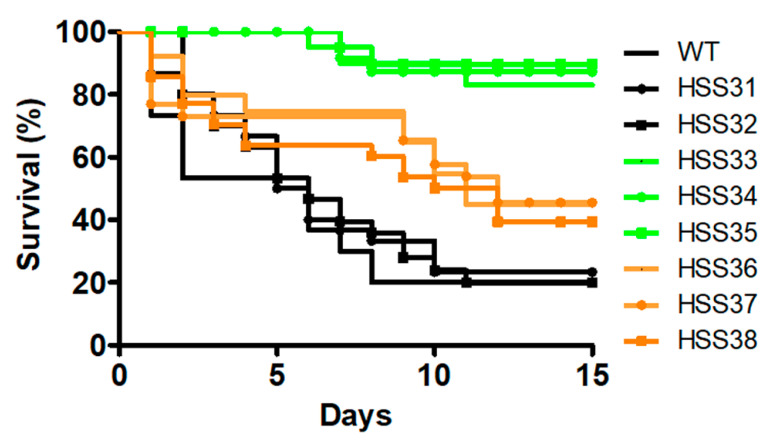
*Galleria mellonella* killing curves by *Sporothrix schenckii* wild-type, control, and *ROT2*-silenced strains. Animal groups contained 30 larvae and were inoculated with 1 × 10^5^ yeast-like cells of the different strains, and survival was recorded daily for two weeks. Data are shown in Kaplan–Meier plots. The statistical analysis showed no differences among the WT, HSS31, and HSS32 strains (*p* = 0.57), among HSS33, HSS34, and HSS35 (*p* = 0.88), or among HSS36, HSS37, and HSS38 (*p* = 0.79). However, the two groups of silenced strains showed curves statistically different when compared between them or with the control group (in black lines; *p* < 0.05, for both cases).

**Figure 8:**
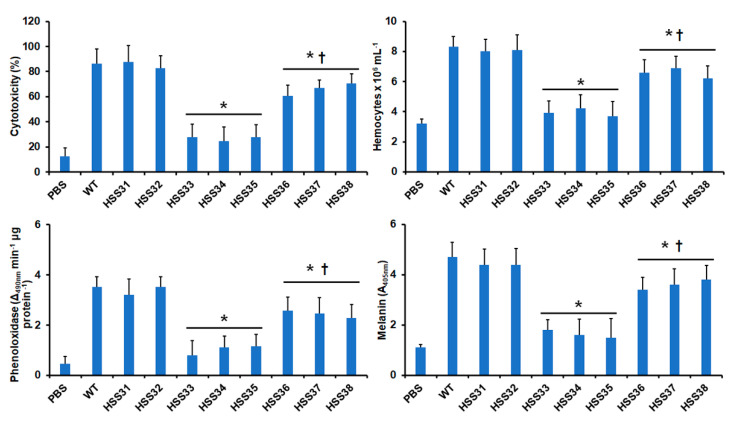
Cytotoxicity and immunological parameters of *Galleria mellonella* larvae infected with different strains of *S. schenckii*. *G. mellonella* larvae were infected with the different fungal strains as described in the Materials and Methods section and incubated for 24 h at 37 °C before animal decapitation and hemolymph collection. The cell-free hemolymph was used to measure lactate dehydrogenase activity, an indicator of cytotoxicity. The 100% cytotoxicity was the enzyme activity measured in lysate hemocytes. The other three parameters were quantified in anticoagulated total hemolymph. PBS, animal group inoculated only with PBS. Data represent means ± SD from ten animals per group, assayed by duplicate. * *p*< 0.05 when compared to wild-type (WT) strain. ^†^ *p* < 0.05 when compared to strains HSS33, HSS34, or HSS35.

The *Sporothrix* virulence was assayed in the thoroughly validated alternative model of systemic sporotrichosis in *G. mellonella* larvae [11,40,70,75,78,79]. Upon inoculation of the fungal load, animal death was recorded on a daily basis for two weeks. The WT and control strains showed similar mortality curves and killed an average of 76.6 ± 0.5% of the animal population with a median survival of 5.6 ± 0.3 days (Figure 7). The strains with intermediate silencing levels were unable to kill the animal population, killing 6.8 ± 1.2% of larvae with a median survival higher than 15 days (Figure 7). However, the high *ROT2* silencing partially restored the fungal ability to kill animals; the three strains killed 57.6 ± 3.2% of the animal population with a median survival of 11.6 ± 1.2 days (Figure 7). When the fungal load isolated from the hemolymph of infected larvae was analyzed, similar colony-forming units were observed for all the WT, control, and silenced strains (average 2.7 ± 0.7 × 10^5^ cells) indicating all the strains had the same ability to adapt and grow in the host milieu. The cell-free lactate dehydrogenase activity in hemolymph has been previously used as a cytotoxicity marker in *G. mellonella* [11,68,69,75], and as expected, minimal levels of this enzyme activity were found in animals injected only with PBS (Figure 8). Cytotoxicity was significantly higher in the animal group inoculated with the WT strain, and the groups infected with the control strains showed similar cytotoxicity levels (Figure 8). However, the intermediate-silenced strains HSS33-HSS35 showed a reduction in the cytotoxicity, and this increased again in the highly *ROT2*-silenced strains HSS36-HSS38 but was lower than those observed in the group infected with the WT strain (Figure 8). Hemocytes, melanin production, and phenoloxidase activity are cellular and humoral elements of insect immunity that are affected during the interaction with fungal pathogens, including *Sporothrix* spp. [11,68,69,71,75,80,81,82]. Hemocyte levels in hemolymph were higher and similar in the WT and control strains HSS31 and HSS32, and these were significantly lower in the intermediate-silenced strains HSS33-HSS35 (Figure 8). The increment in *ROT2* silencing associated with strains HSS36-HSS38 partially restored the hemocyte levels (Figure 8). A similar trend was observed for both phenoloxidase activity and melanin production, with high and similar levels for both parameters in the animal groups infected with the WT and control strains, low levels in the animal groups infected with strains HSS33-HSS35, and a partial restoration of enzyme activity and pigment production in the animal groups infected with the strains HSS36-HSS38 (Figure 8). Collectively, these data indicate that *ROT2* silencing affected the interaction of *S. schenckii* with *G. mellonella* larvae.

## 4. Discussion

*S. schenckii* is part of the *Sporothrix* pathogenic clade, and its basic and biological aspects are the most studied among the causative agents of sporotrichosis [3]. Similar to other fungal species, the study of the fungal cell wall, in terms of synthesis, composition, organization, and dynamics results, is essential to understand most of the details of the host–fungus interplay [12,13,14,83]. The *N*-linked and *O*-linked glycans covalently attached to cell wall proteins are in most cases species-specific oligosaccharides, with unique structures and compositions that generate particular molecular barcoding that influences the interaction with the host immunity [26,84,85]. For the case of *S. schenckii*, peptidorhamnomannan is a signature heterogeneous molecule that contains both *N*-linked and *O*-linked glycans [22], which have a relevant role during the *Sporothrix*–host interaction [11,22,23,63,74,75,86]. Glycosyl hydrolases participating in the *N*-linked glycosylation pathway, in particular glucosidase II, have been thoroughly studied in commensal and pathogenic yeast species, such as *C. albicans*, *Candida glabrata*, and *Saccharomyces cerevisiae* [35,36,87,88]; however, little is known about this enzyme activity and its relevance during the interaction of filament fungal species with the host. Currently, the study of α-glucosidase II and the protein glycosylation pathways has been focused on the optimization of protein secretion for biotechnological purposes [88,89].

In *S. cerevisiae* and *C. glabrata*, no growth defect was observed upon *ROT2* disruption [35,44,87], contrasting with the sick phenotype reported for the *C. albicans rot2*Δ null mutant, where cells tended to aggregate and growth rate was significantly affected [36]. Here, *S. schenckii ROT2* silencing did not affect growth or morphology, behaving like the *S. cerevisiae* system. These results are in line with the previous observation that the heterologous expression of *S. schenckii ROT2* was capable of fully restoring the phenotype of the *S. cerevisiae rot2*Δ null mutant strain but partially the phenotype of a *C. albicans rot2*Δ null mutant strain [44]. In addition, this supports the previously generated hypothesis to explain these differences, related to the lack of transmembrane domain and classic ER-retention motive in the *S. schenckii* enzyme and therefore dependence on the beta subunit to remain attached to the ER membrane, a situation dispensable for *C. albicans* enzyme [44]. The measurement of enzyme activity with the fluorogenic substrate in the presence of castanospermine, along with the detection of the *N*-linked glycan core on the cell wall, was an indication that the gene silencing negatively affected the α-glucosidase II activity. The presence of the *N*-linked glycan core on the cell surface suggested that the *N*-linked glycan outer chain was not synthesized, and these short *N*-linked glycans are responsible for the lower levels of *N*-linked glycans found at the cell surface. A similar observation was reported for the *C. albicans rot2*Δ null mutant [36], underlining the importance of proper processing of the *N*-linked glycan core by α-glucosidase II to allow elongation of the outer chain in the Golgi complex. Interestingly, the reduction in α-glucosidase II positively affected the *O*-linked glycan levels. This observation is similar to that reported previously in the *S. schenckii OCH1* silenced strain [40] and reinforces the hypothesis that sites of *O*-linked glycosylation are covered by the bulky *N*-linked glycan chain, and once this outer chain is removed, these sites become accessible for protein-*O*-mannosyltransferases to initiate the *O*-linked glycosylation [40]. Alternatively, since the silenced mutants showed increased levels of cell wall protein, it is possible to hypothesize these are richer in *O*-glycosylation sites than the proteins found in the WT cell wall. This hypothesis implies that the differences are in both the quantity and quality of polypeptides and this aspect remains to be addressed. Nevertheless, the compensatory effect restored the cell wall mannose content to WT levels, suggesting that covering the cell surface with mannose-based glycans is required for *S. schenckii*.

As reported in *C. albicans* and *S. cerevisiae*, the cell wall was affected in *S. schenckii* cells with reduced α-glucosidase II activity. Like in these two yeast species [36,90], here, *ROT2* silencing led to an increment in chitin content, but contrary to the finding reported in those models, where lower glucan content was observed in the cell wall [36,91], the *S. schenckii ROT2* silencing led to higher glucan levels. This increment in glucan content was only observed in the cells with intermediate *ROT2* silencing levels and was associated with lower mannose and rhamnose content. A similar observation was recently reported by our group during the *S. schenckii RlmD* silencing; these cells showed low levels of rhamnose and high glucan content at the cell wall [11]. Thus, it is possible to hypothesize that a reduction in rhamnose content leads to compensatory increments in glucan levels. In line with this observation, the *S. globosa* cell wall naturally has low rhamnose levels and high glucan content [75]. As mentioned, the apparent normal rhamnose and mannose composition in the highly *ROT2*-silenced strains is explained by the compensatory increment in the *O*-linked glycans found at the cell wall. All these cell wall changes affected the wall organization, and the structural polysaccharides β-1,3-glucan and chitin, normally found in the inner layer of the cell wall [20], were exposed at the cell surface. Similar observations have been reported in *S. schenckii* silenced strains and *Candida* null mutant strains with defects in the protein glycosylation pathways [11,26,40,50,60,62,73,92,93,94].

In terms of interaction with human PBMCs, the WT strain replicated the previously reported results in terms of PRR dependence for cytokine production, confirming that MR is dispensable for the stimulation of the analyzed proinflammatory cytokines, but not for IL-10 [20], and a strong dependency on dectin-1 for cytokine stimulation [11,20,40]. Similar to other reports, CR3, TLR2, and TLR4 played also a significant role in the stimulation of TNFα and IL-6 [11,20,40,86]. The reduced levels of both cytokines stimulated by the strains with intermediate *ROT2* silencing levels can be explained by the fact that the CR3 and TLR4 ligands, named peptide rhamnomannan and rhamnose, respectively [11,86], were absent or reduced in the wall of these cells, which fits with our cell wall analysis. Interestingly, these cells showed increased β-1,3-glucan content and exposure at the cell wall, and this was not sufficient for a strong dectin-1-dependent cytokine stimulation, suggesting that proper TNFα and IL-6 stimulation requires the collaborative stimulation of dectin-1 along with TLR4 or CR3. Similar observations have been reported for *C. albicans* and *Candida parapsilosis* [60,92,95]. The restoration of TNFα and IL-6 levels in the highly *ROT2*-silenced strains reinforces the hypothesis that both mannose and rhamnose-based glycoconjugates work in collaboration with β-1,3-glucan to stimulate these cytokines. Since it was reported that *S. schenckii* chitin can stimulate proinflammatory cytokine production [96], an alternative explanation for these results is that the increased levels of cell wall chitin, which was more accessible to its receptor than the polysaccharide found on the cell surface of the WT cells, is responsible for this observation. For the case of IL-1β and IL-10, our result clearly shows that the strong cytokine production by the silenced strains was driven by dectin-1 engagement with its ligand, and the other receptors were dispensable for this stimulation. Similar results have been reported previously for *S. schenckii* [11,20,40,86]. Our observations about IL-1β contrast with those previously reported [86], where stimulation of this cytokine was dependent also on MR and CR3. This discrepancy is likely related to the cell preparation; here, live cells were used to stimulate cytokine production, while in the previous report, heat-killed cells were used, which have a different cell wall organization than the live ones and, as a consequence, different ability to stimulate cytokines [20,50,54,60,73,83,97]. Once again, chitin recognition might be involved in this increased ability to stimulate this cytokine, but this remains to be addressed.

The interaction with human monocyte-derived macrophages was also affected by *ROT2* silencing. The mutant strains with intermediate silencing levels tended to show a reduction in the ability to be phagocytosed, which is in line with the previous reports using *S. schenckii OCH1* silenced strains [40]. Silencing of any of these two genes is associated with a reduction in rhamnose content [40], which suggests that this sugar moiety could be involved in the uptake by these fungal cells. This is supported by the loss of the blocking effect of anti-TLR4 antibodies in the phagocytosis assays with the strains HSS33-HSS35, as rhamnose has been proposed as the ligand of this receptor [11]. Since the cell wall of these mutant cells was not fully depleted of rhamnose- and mannose-based glycoconjugates, it was possible to observe also the contribution of CR3 and MR to the internalization event, as previously reported [63]. Nevertheless, in the case of both the intermediate and highly *ROT2*-silenced strains, dectin-1 plays a significant role in the uptake process, similar to the interaction of these immune cells with *C. albicans* [98]. However, the dectin-1-β-1,3-glucan interaction is not the sole ligand–receptor interaction needed to support *S. schenckii* phagocytosis, as this cell wall component was higher in the intermediate-silenced strains and equally exposed at the cell surface, and uptake was not higher or at least at the WT level, suggesting that TLR4 signaling is as relevant as the one triggered by dectin-1 activation. The increased uptake observed with the strains HSS36-HSS38 is likely explained by the increased levels and exposure of chitin at the cell surface since it was reported that this cell wall polysaccharide promotes *S. schenckii* uptake by macrophages [96].

The *ROT2* silencing affected *S. schenckii* virulence, and this observation is in line with the *ROT2* disruption in *C. albicans* and *C. glabrata* [36,87], indicating that proper processing of the *N*-linked glycan core is also required for the *S. schenckii* ability to kill the host. Since similar fungal burdens were recovered from all the strains, it is unlikely that results are biased by the inability of the silenced strains to adapt and grow in the host tissues. Instead, our results point out defects in the virulence factor repertoire, in particular those that depend on proper glycosylation to display its molecular function. Among the virulence factors found in *S. schenckii* [99], adhesins are factors that are likely affected by defects in the protein glycosylation, since some of them are heavily glycosylated proteins, containing mannose- and rhamnose-based oligosaccharides [23,100,101]. Alternatively, the classic secretory pathway is linked to proper protein glycosylation, and defects in these are likely to affect protein secretion, including secreted proteases and lipases [102].

In conclusion, *S. schenckii ROT2* is required for proper *N*-linked glycosylation and cell wall organization and composition. The silencing of this gene affected the interaction of this fungus with human PBMCs and monocyte-derived macrophages, and led to virulence attenuation.

## Figures and Tables

**Table 1:** α-Glucosidase activity and analysis of the *N*-linked glycan core in wild-type, control, and *ROT2*-silenced mutant strains.

Strain	α-Glucosidase Activity ^a^	α-Glucosidase Activity ^a^ + 10 μM Castanospermine	Glc_2_Man_9_GlcNAc_2_ (ng 10^9^ Cells^−1^) ^b^	Glucose (ng 10^9^ Cells^−1^) ^c^
Wild-type	1126.8 ± 106.5	598.4 ± 124.1	No detected	No detected
HSS31	1052.2 ± 88.4	626.5 ± 101.5	No detected	No detected
HSS32	1103.1 ± 124.6	601.4 ± 92.8	No detected	No detected
HSS33	869.4 ± 101.2 *	395.1 ± 79.5 *	33.3 ± 26.4 *	2.4 ± 0.8
HSS34	836.5 ± 98.4 *	358.0 ± 94.4 *	39.7 ± 22.1 *	1.9 ± 0.6
HSS35	856.6 ± 72.4 *	362.8 ± 83.4 *	34.8 ± 12.6 *	1.7 ± 0.9
HSS36	614.7 ± 88.7 **	7.4 ± 3.2 **	105.3 ± 25.4 **	9.2 ± 2.2
HSS37	580.7 ± 118.6 **	6.1 ± 4.1 **	102.6 ± 18.7 **	11.4 ± 1.4
HSS38	621.7 ± 110.1 **	8.3 ± 3.7 **	96.5 ± 20.4 **	10.1 ± 1.7

^a^ Expressed as nmoles of 4-methylumbelliferone released per min per total protein. ^b^ Endoglycosidase H-trimmed glycans from the cell wall analyzed by high-performance anion-exchange chromatography with pulsed amperometric detection. ^c^ The endoglycosidase H-released material was hydrolyzed with recombinant α-glucosidase before analysis by high-performance anion-exchange chromatography with pulsed amperometric detection. * *p* < 0.05 when compared to wild-type, HSS31, HSS32, HSS36, HSS37, or HSS38 strains. ** *p* < 0.05 when compared to wild-type, HSS31, HSS32, HSS33, HSS34, or HSS35 strains.

**Table 2:** Cell wall protein content, porosity, and ability to bind Alcian blue of *Sporothrix schenckii* wild-type, control, and *ROT2*-silenced strains.

Strain	Protein (μg mg Cell Wall^−1^)	Porosity (%) ^a^	Alcian Blue Bound (μg OD_600nm_ = 1.0^−1^)
Wild-type	192.8. ± 27.2	81.6 ± 7.7	115.9 ± 11.5
HSS31	183.9 ± 33.4	78.5 ± 5.5	121.1 ± 13.6
HSS32	199.2 ± 38.2	77.3 ± 6.2	124.0 ± 8.8
HSS33	286.3 ± 47.2 *	98.2 ± 6.6 *	83.4 ± 9.3 *
HSS34	295.1 ± 39.8 *	97.5 ± 4.9 *	88.4 ± 12.5 *
HSS35	279.2 ± 22.5 *	98.6 ± 7.1 *	79.3 ± 7.8 *
HSS36	298.9 ± 31.1 *	70.1 ± 5.2	111.9 ± 12.3
HSS37	295.0 ± 42.1 *	77.9 ± 6.3	128.1 ± 14.6
HSS38	276.4 ± 33.4 *	83.5 ± 7.0	123.4 ± 16.5

^a^ Relative to diethylaminoethyl-dextran and normalized against poly-L-lysine. * *p* < 0.05 when compared to wild-type, HSS31, HSS32, HSSS36, HSS37, or HSS38 strains.

## Data Availability

Not applicable.

## Supplementary Materials

The following supporting information can be downloaded at: https://www.mdpi.com/article/10.3390/jof8111220/s1, Figure S1. Cytokine stimulation by human peripheral blood mononuclear cells stimulated with Sporothrix schenckii wild-type, control, and ROT2-silenced strains. Human peripheral blood mononuclear cells were preincubated for 60 min with 10 μg mL^−1^ of irrelevant IgG1, IgG2, or IgG2ak antibodies and then coincubated with yeast-like cells for 24 h. In all cases, the interactions were centrifuged, supernatants collected, and cytokines quantified by ELISA. None, refers to the system where human cells were preincubated with PBS and then challenged with the fungal cells. Control refers to wells where the human cells were preincubated only with PBS. Results are the media ± standard deviation from data generated with samples from eight donors, analyzed by duplicate.
