# Supplementary material for: Silencing of ROT2, the Encoding Gene of the Endoplasmic Reticulum Glucosidase II, Affects the Cell Wall and the Sporothrix schenckii–Host Interaction
Source: J Fungi (Basel). 2022 Nov 18;8(11):1220. doi: 10.3390/jof8111220 (PMC9692468; doi:10.3390/jof8111220)
Supplement: Supplementary file 1 [file jof-08-01220-s001.zip › jof-1969086-supplementary.pdf]

Journal of *Fungi*

MDPI

Supplementary Material

Silencing of *ROT2*, the Encoding Gene of the Endoplasmic Reticulum Glucosidase II Affects the Cell Wall and the *Sporothrix schenckii*-Host Interaction

Luz A. López-Ramírez [1], Iván Martínez-Duncker [2], Anayeli-Márquez-Márquez [1], Ana P. Vargas-Macías [1] and Héctor M. Mora-Montes [1,*]

[1] Departamento de Biología, División de Ciencias Naturales y Exactas, Campus Guanajuato, Universidad de Guanajuato, Noria Alta s/n, col. Noria Alta, C.P., Guanajuato Gto. 36050, Mexico;

[2] Laboratorio de Glicobiología Humana y Diagnóstico Molecular, Centro de Investigación en Dinámica Celular, Instituto de Investigación en Ciencias Básicas y Aplicadas, Universidad Autónoma del Estado de Morelos, Cuernavaca Mor. 62209, México;

* Correspondence: hmora@ugto.mx; Tel.: +52-473-732-0006 (ext. 8193)

1. Supplementary Figure

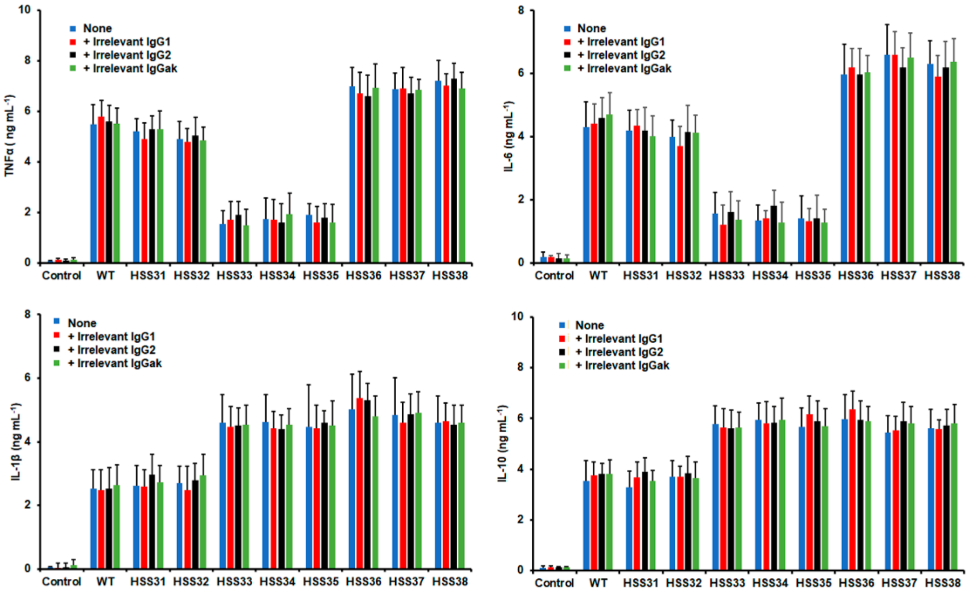

Figure S1. Cytokine stimulation by human peripheral blood mononuclear cells stimulated with *Sporothrix schenckii* wild-type, control, and *ROT2*-silenced strains. Human peripheral blood mononuclear cells were preincubated for 60 min with 10 µg mL^{-1} of irrelevant IgG1, IgG2, or IgG2ak antibodies and then coincubated with yeast-like cells for 24 h. In all cases, the interactions were centrifuged, supernatants collected, and cytokines quantified by ELISA. None, refers to the system where human cells were preincubated with PBS and then challenged with the fungal cells. Control refers to wells where the human cells were preincubated only with PBS. Results are the media ± standard deviation from data generated with samples from eight donors, analyzed by duplicate.
